# Determinants of the need for respite according to the characteristics of informal carers of elderly people at home: results from the 2015 French national survey

**DOI:** 10.1186/s12913-021-06935-x

**Published:** 2021-09-21

**Authors:** Wilfried GUETS, Lionel PERRIER

**Affiliations:** 1 Univ Lyon, Université Lumière Lyon 2, GATE UMR 5824, F-69130 Ecully, France; 2 Univ Lyon, Léon Bérard Cancer Center, GATE UMR 5824, F-69008 Lyon, France; 3 Human and Social Sciences Department, Léon Bérard, F-69008 Lyon, Centre France

**Keywords:** Econometrics, Health status, Informal carers, Respite

## Abstract

**Background:**

The demographic and social changes associated with population aging and the increasing incidence of chronic diseases underscore the importance of the role of informal carers. The number of informal carers is increasing and negative consequences associated with providing care, such as burnout, are known. However the influence of socioeconomic and psychological factors on the need for respite have not been well characterized to date. Informal care represents an essential component of health care systems and long-term care. The purpose of this study was to shed light on how the characteristics of informal carers affect the need for respite.

**Methods:**

We used data from a nationally representative survey, Capacités Aides et Resources des Seniors (CARE - ménage), collected in 2015 by the National Institute for Statistics and Economic Studies (INSEE) and the Directorate for Research, Studies, Assessment and Statistics (DREES). The determinants of the need for respite among the characteristics of informal caregivers were explored using a probit model. To handle missing data, sensitivity analyses were performed using multiple imputations.

**Results:**

Our study included *N* = 4033 dyads of informal carers and care recipients. The mean age was 61 for carers. The majority of carers were female, married, the child of the care recipient. Almost 27% reported a need for respite. A worse health status, feeling of loneliness, having a lack of time for oneself and needing to provide more than 30 h of care per month very significantly increased the need for respite irrespective of whether or not the carer lived with the care recipient (*p* < 0.01). Providing care to other persons was likely to induce a greater need for respite (*p* < 0.01). Cohabitation of the informal carer and the care recipient was likely to increase the need for respite (*p* < 0.05). Conversely, however, being closely acquainted with the care recipient showed a reduced need for respite in comparison with that of carers who are married to their care recipient (*p* < 0.05).

**Conclusions:**

These findings provide useful information for policymakers, physicians and other health professionals for reducing carers’ risk of exhaustion and burnout and for referring carers to the relevant service, e.g. psychological intervention, respite care support, training support and education support.

**Supplementary Information:**

The online version contains supplementary material available at 10.1186/s12913-021-06935-x.

## Introduction

Informal carers represent unpaid persons such as family members, neighbours, close acquaintances or other significant individuals who provide daily assistance to a family member or dependent elderly person who cannot care for himself or herself. Informal care represents an essential component of health care systems and long-term care, and a significant proportion of the population dedicates a particular part of their time to providing care to care recipients (parents, children or spouses). In France, almost 3.9 million informal carers of older adults (aged 60 or more) living at home provide regular care due to age or a health problem [1]. Informal carers have a ubiquitous and very substantial presence throughout the world. International Alliance of Carer Organizations (IACO) provided the following figures of informal carers internationally: 43.5 million in the USA (2015), 8.1 million in Canada (2012), 6.5 million in the UK (2011) and more than 8 million people in France (2019) [2]. Given the situation marked by the increase in expenditure for the health system in the majority of western countries due to ageing populations, the demand for informal care is likely to increase over the coming decades [3].

In France, according to the projections of the National Institute for Statistics and Economic Studies (*INSEE*), the proportion of people aged over 60 years will increase sharply until 2035 [4]. This sharp increase will be transitory and will correspond to the “baby-boomer*”* generation ageing. In 2015, 3 million people aged 60 or older living at home reported being regularly assisted with activities of daily living because of their age or a health condition [5]. Simultaneously, among carers, the number of individuals likely to be able and willing to provide care has probably reduced as a result of a range of socio-cultural trends, such as demographic changes, the increase of female participation in the labour force, cultural values and changes to family structures [6–10]. Therefore, demographic and social changes associated with population ageing have resulted in much debate regarding how care is provided to the elderly and/or people with disabilities [11, 12].

Informal carers play a strategic role in the daily activities of their dependent care recipients. Although some carers view care provision as propitious and a generator of positive utility, it can be seen that for some informal carers, care provision has lost these qualities [13–16]. When this happens, providing informal care produces negative consequences for carers as a result of a high risk of exhaustion (strain/burnout) if carers do not receive external assistance. Generally, informal care negatively affects the carer’s work productivity [17–22] and their health [23–27]*.* Despite rapid impairments being observed in situations involving an overwhelming burden, there is more concern regarding the gradual worsening of carers’ quality of life [28–30]. In light of this, it is clear that many carers need support services to improve their health and quality of life [31]. This need is substantial for carers with a high risk of exhaustion, who remain without support at their disposal. As a result, the consequential situation may potentially lead to a “double boomerang” effect of one care recipient receiving informal care leading to two dependent individuals using formal care [25].

Assuming that carers occupy an ambiguous position within the social care system [32], the majority of services are predominantly structured around recipients. Nevertheless, many support services dedicated to carers have been developed across countries [33, 34], particularly respite care [35, 36]. Respite care generally provides temporary relief to informal carers from continuing caregiving responsibilities and restores resilience and improves the quality of life and well-being of carers [37]. The need and the claims for respite assistance are priority considerations in the debate regarding the prevention of frailty of the carers. Despite the rapid intensification of focused carer support programmes in recent decades, due to the increasing number of carers over this period [38], there are still many who become overwhelmed with the burden of providing informal care. Several studies have found inadequacy of services on offer, ambivalence in carers’ attitudes, a lack of available time [39] and a feeling of guilt when carers request assistance [40, 41]. Given the potential benefit of respite and the gradual recognition of this fact by health professional and policymakers, the assessment of respite programmes establishes that the timing at which services were offered and then subsequently used by carers was deemed both “too late” [42] and “too little” [43], even for overburdened carers [44]. Nevertheless, despite the low use of respite, many carers reported a significant need in general [45, 46] and particularly for day care [45, 47].

Regarding this significant public health issue, it is essential to identify and understand factors influencing the need for respite among carers, such health status [48]. Note that, the need for respite does not necessarily equal the need for respite care [49].

In this paper, we investigated the need for respite related to the characteristics of carers and recipients. Our paper constitutes an important contribution to the field because it provides a first exploratory analysis taking advantage of the large and recent French national data set and a broad pattern of explanatory variables.

## Material and methods

### Data source

Data used in our study stemmed from the *Capacités Aides et Resources des Seniors (CARE ménage)* [50], a nationally representative survey carried out in France’s metropolitan areas in 2015 by the National Institute for Statistics and Economic Studies *(INSEE)* and the Directorate for Research, Studies, Assessment and Statistics (*DREES)*. We used both the data of carers [51] and care recipients [52]. The carer survey (*CARE ménage - Volet “Aidants”)* was a supplementary section of *CARE ménage - Volet “Senior”*, collected in 2015. The survey protocol favoured face-to-face data collection. The survey was conducted by phone when face-to-face contact was not possible.

The *CARE* survey focuses on the living conditions of people aged 60 or over living at home, their difficulties in carrying out the activities of daily living and the assistance they receive. About 15,000 older people were interviewed, including healthy people. A total of 10,628 care recipients among the elderly were included in the study. Informal carers represented 6201 (16 years of age or older) of those declared by recipients.

Regarding the need for respite (dependent variable), the carer had to answer “yes”/“no” to the following question: *“Do you need respite? / Do you need more respite for longer periods of time?”.* The characteristics, technical details and a full description of the *CARE* survey are available in the technical notice of the *DREES* website [53, 54].

## Methods

We used descriptive statistics to provide details on the study sample, including informal carers and care recipients. We used multivariate regressions to explore variables influencing the need for respite. We assumed that the need for respite depends on the health status, living arrangement and various socio-economic characteristics. Hence, we selected a number of variables based on scientific knowledge and existing literature [24, 25, 48, 55–57].

We modelled the *Need for respite*_*i*_ (dichotomous dependent variable) through a *probit* model (primary analysis). The econometric specification of the *Need for respite*_*i*_ in was formulated as follows:

1 $$ Need\ {for\ respite}_i={\beta}_0+{\beta}_1{H}_i+{\beta}_k{X}_i+{\varepsilon}_i $$

Where *H*_*i*_ stands for the health status of the carers. *X*_*i*_ represents explanatory variables related to socio-economic dimensions of the characteristics of informal carers and recipients. *β*_*k*_ represents the parameters to be estimated and *ε*_*i*_ represents the error term. The dependent variable *Need for respite*_*i*_ was defined as:

2 $$ Need\ {for\ respite}_i=\left\{\begin{array}{c}1\  if\ Need\ {for\ respite}_i>0\\ {}0\  if\ Need\ {for\ respite}_i\le 0\end{array}\right. $$

We pay particular attention to the interest variable “health status”. It referred to a set of levels describing different carer health statuses: very good; good; fair; bad; very bad. We assumed that a worse health status induces more need for respite. The other characteristics included in the analyses were: (i) length of time for care provision (in year), income, marital status, filiation, care for other persons, cohabitation, replacement in case of unavailability, age, gender, duration of care provision (in hour per month), use of support group, and of training for informal carers; (ii) and number of chronic diseases, use of formal care, restrictions for activities of daily living (ADL) and Instrumental activities of daily living (IADL), Mental health index (MHI5) for care recipients. After we checked no high correlation was identified between included predictors, a logistic regression was performed with the need for respite as dependent variables.

Furthermore, we performed a secondary analysis for robustness checks by using a composite index as a proxy of the health status (interest variable) of the informal carers. In order to avoid problems of multicollinearity, we deemed appropriate to analyse them separately. Therefore, we built a Health Status Composite Index (HSCI) capturing the level of vulnerability related to negative consequences of the carers’ health states [58], considered to be the interest variable. In keeping with the literature, the following as indicators were retained: stress, anxiety, back problems, physical exhaustion, sleep disorders. The HSCI, which reflects a linear combination of such indicators, can also be assumed as a subjective burden of informal care. These selected variables were turned into the HSCI by computing the Principal Components Analysis (PCA), which amounts to a substantial contribution to the main component. The PCA was related to the strain of carers (this comprised high values for the most affected), with cumulative inertia in the first axis. We used *Varimax* rotation to change the PCA coordinates that maximise the variances of the sum of the squared loadings. Thus, all of the coefficients of each component became either large or close to zero, with few intermediate values. The goal was to capture the association of each variable with at the most one factor. As we only considered the first factor/axis, the composite index provided substantial information regarding retained variables (stress, anxiety, back problems, physical exhaustion, sleep disorders). Finally, the econometric specification of the secondary analysis of the *Need for respite*_*i*_ was determined as follows:

3 $$ {Need}_i={\beta}_0+{\beta}_1{HSCI}_i+{\beta}_k{X}_i+{\varepsilon}_i $$

Where *HSCI*_*i*_ represents the Health Status Composite Index capturing the carer vulnerability.

All variables and parameters remained unchanged compared with primary analysis, except for the variable “health status” which was replaced by the “HSCI” variable.

To deal with the problem of multicollinearity, overfitting and reduce the number of predictors, we performed a stepwise backward selection with *p* < 0.05 in order to determine which among socio-demographic characteristics significantly affect the need for respite. We have followed the recommendations of MA Babyak [59]. Only predictors with *p*-value < 0.05 were included in the multivariate analysis after the stepwise backward selection [60].

To handle missing data in primary and secondary analyses, sensitivity analyses were performed using multiple imputations (m = 10 imputations) with chained equations [61, 62]. The data after multiple imputations replaced the missing observations on the covariates of the full model. The results of multiple imputations were provided using all covariates.

The level of significance of 0.05 was retained. We carried out all the statistical analyses with STATA SE-64 Statistical software 16 (StataCorp. LP, College Station, TX, USA).

## Results

### Descriptive statistics

**Fig. 1:**
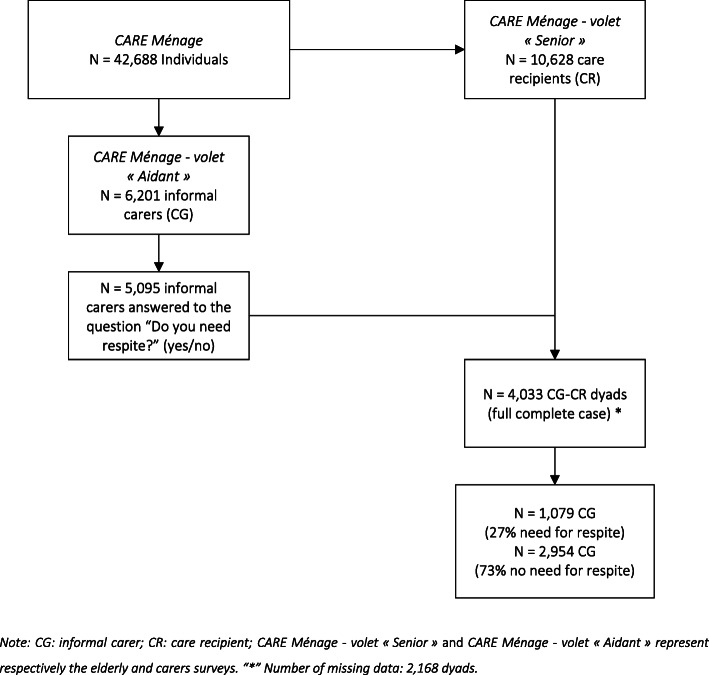
Study population

Figure 1 describes the entire study population. The *CARE* survey contains 42,688 individuals, among which *N* = 6201 were informal carers and *N* = 10,628 were care recipients. Carers’ data were then matched with recipients’ data to provide a dyadic sample study. Of those, only *N* = 5095 informal carers reported the need for respite (yes/no). Finally, our study included *N* = 4033 informal carer-care recipient dyads with the full complete case. Data was missing in *N* = 2168 dyads (i.e. 6201 - 4033) and calculated with imputations.

Table 1 provides details of the characteristics of informal carers and care recipients for the entire included population and for informal carers who did and did not need respite. Of 4033 informal carers, almost 62% reported a health status of “very good” or “good”. Almost 66% of carers were married. The mean age of carers was 61 years (SD ± 14) with a range of 18–96. Female carers accounted for 61%. Almost 41% of carers lived in cohabitation. The majority of carers (54%) were offspring of those for whom they were caring, 28% partner by marriage, 13% a family member and 5% a close acquaintance. Almost 77% reported the possibility of replacement in case of unavailability. Almost 58% of carers reported a length of time for care provision greater than 5 years. Informal carers who provided care for less than 30 h per month represented 45%. Regarding the need/use for support services, 27% of informal carers needed respite while 11% used training services and 4% support groups.

**Table 1:** *Characteristics of informal carers and care recipients*

	Entire included population (*N* = 4033)	Needed respite (*N* = 1079)	Did not need respite (*N* = 2954)	Test of independence *p*-value^e^
Informal carers’ characteristics				
Health status^a^ %				
Very good and good	62	49	67	0.000
Fair, bad and very bad	38	51	33	
Income (monthly, before tax)^b^ %				
< 1800€	29	29	29	0.721
≥ 1800€	71	71	71	
Marital status %				
Single	19	19	19	0.709
Married	66	66	65	
Divorced or Widowed	15	15	15	
Mean age (SD)	61 (14)	63 (13)	60 (14)	0.000 ^*f*^
Sex %				
Female	61	67	59	0.000
Male	39	33	41	
Cohabitation %	41	56	35	0.000
Filiation %				
Partner by marriage	28	37	25	0.000
Child^c^	54	52	55	
Family member	13	10	14	
Close acquaintance	5	1	6	
Care for other persons	21	22	21	0.351
Replacement in case of unavailability	77	68	81	0.000
Length of care provision^d^ %				
< 1 year	4	5	4	
1–5 years	38	37	39	0.484
≥ 5 years	58	58	57	
Duration of care provision (per month) %				
< 30 h	45	20	54	
30 h–60 h	17	16	17	0.000
60 h–150 h	21	29	18	
> 150 h	17	34	11	
HSCI^g^, mean (range)	0.21(−1.5 to 5.9)	1.7(−1.4 to 5.9)	−0.34(−1.5 to 5.9)	0.000^f^
Stress and anxiety %	36	60	26	0.000
Back problems %	24	43	16	0.000
Physical exhaustion %	33	63	22	0.000
Sleep disorders %	23	43	15	0.000
Feeling of loneliness %	41	67	32	0.000
Problem of lack of time %	35	79	18	0.000
Need for respite %	27	–	–	–
Use of support group %	4	6	3	0.000
Use of training %	11	20	8	0.000
Care recipient characteristics				
Health status^a^ %				
Very good and good	15	8	18	
Fair, bad and very bad	85	92	82	0.000
Mean age (SD)	81(9)	81(10)	81(9)	0.09^f^
Sex %				
Female	68	63	70	0.000
Male	32	37	30	
Number of diseases				
One disease or less	33	31	33	0.094
More than one disease	67	69	67	
ALD restrictions				
At least one %	20	20	21	0.792
Number (0–6), mean (SD)	0.47(1.15)	0.47(1.17)	0.48(1.14)	0.937^f^
IALD restrictions				
At least one %	68	67	69	0.314
Number (0–7), mean (SD)	1.8(1.9)	1.8(1.9)	1.8(1.7)	0.998
MHI-5, mean (SD)	66(22)	67(21)	66(22)	0.213
Use of formal (professional) care	35	35	35	0.983

Notes: *SD* standard deviation; ^a^ Health status: 5 categories recoded into two categories; ^b^ Income level: 5 categories recoded into two categories; ^c^ Child: recoded item as daughter or son; ^d^ Length of care provision: categories recoded into three categories; ^e^ Chi2 statistical test; ^f^ Test of the difference of the means;^g^ The HSCI varies from − 1.5 to 5.9, where the highest value represents a situation of strain and important burden for informal carers. Also, the correlation coefficient between the health status and HSCI was 0.403. It is not impossible that using them both variables in the same model could increase the risk of multicollinearity. ^h^ *MHI-5* Mental Health Inventory (0–100), with 100 representing the score of optimal mental health. (I)ALD (Instrumental) Activity of Daily Living

Regarding care recipients, the majority (85%) reported quite bad health status and 68% were female. The mean age was 81 years (SD ± 9) with a range of 60–107 and 67% were suffering from more than one chronic disease. Almost 20% of recipients were assisted for at least one ADL, whereas 68% of recipients were assisted for IADL. A total of 35% care recipients received formal care.

### Econometric modelling

#### Primary analysis

Table 2 reports the results of the econometric modelling for the primary analysis. We reported the model with complete cases (1), after stepwise backward selection (2) and with multiple imputations (3). Our findings show that the poorest health status for informal carers significantly increased the need for respite (*p* < 0.05). The need for respite was significantly reduced for close acquaintances compared to partners by marriage (*p* < 0.05). Providing care to other persons was likely to induce a greater need for respite (*p* < 0.01). The cohabitation of the informal carer and the care recipient was likely to increase the need for respite (*p* < 0.05). Our results also indicate that the feeling of loneliness (*p* < 0.001), the lack of time (*p* < 0.001), the duration (in hour per month) for care provision (*p* < 0.001), the use of training (*p* < 0.001), and the use of support groups (*p* < 0.01) significantly increased the need for respite. Our results indicated that age was not likely to affect carers’ need for respite at 5% significance level.

**Table 2:** Primary analysis - Determinants of the need for the respite of informal carers

	(1)			(2)			(3)	
	Full model			Model with stepwise backward selection			Full model with multiple imputations	
Variables	Coef.	*p*-value	M.E.	Coef.	*p*-value	M.E.	Coef.	*p*-value
Health Status – (*Very good)*	(ref.)			(ref.)			(ref.)	
Good	0.188*	(0.021)	0.04	0.177*	(0.015)	0.03	0.178**	(0.008)
Fair	0.350***	(0.000)	0.07	0.310***	(0.000)	0.06	0.304***	(0.000)
Bad	0.492***	(0.000)	0.10	0.438***	(0.000)	0.09	0.417***	(0.000)
Very bad	0.690*	(0.013)	0.15	0.645*	(0.016)	0.14	0.608*	(0.019)
Length of time for care - (< 1 year)	(ref.)			–			(ref.)	
1–5 years	−0.106	(0.393)		–			−0.110	(0.350)
> 5 years	−0.135	(0.267)		–			−0.097	(0.434)
Income Level – (< 800€)	(ref.)			–				
800€ - 1200€	0.192	(0.267)		–			0.112	(0.520)
1200€ - 1800€	0.231	(0.133)		–			0.174	(0.272)
1800€ - 2500€	0.244	(0.108)		–			0.120	(0.430)
> 2500€	0.360*	(0.016)	0.07	–			0.286	(0.055)
Marital status – (Single)	(ref.)			–			(ref.)	
Married	−0.048	(0.547)		–			−0.055	(0.452)
Divorced	0.148	(0.292)		–			0.290*	(0.016)
Widowed	−0.114	(0.284)		–			−0.029	(0.769)
Filiation – (Partner by marriage)	(ref.)			(ref.)			(ref.)	
Child	0.159	(0.134)		0.211**	(0.007)	0.04	0.159	(0.087)
Family member	0.186	(0.117)		0.225*	(0.022)	0.05	0.143	(0.164)
Close acquaintance	−0.546**	(0.008)	−0.10	−0.546**	(0.003)	−0.09	−0.617*	(0.010)
Care for other persons	0.165**	(0.010)	0.03	0.174**	(0.003)	0.04	0.144**	(0.008)
Cohabitation	0.193*	(0.019)	0.04	0.177*	(0.014)	0.04	0.145*	(0.043)
Feeling of loneliness	0.380***	(0.000)	0.08	0.369***	(0.000)	0.08	0.366***	(0.000)
Lack of time	1.342***	(0.000)	0.28	1.383***	(0.000)	0.29	1.332***	(0.000)
Replacement in case of unavailability	−0.064	(0.304)		–			−0.125*	(0.032)
Age (CG)	0.026	(0.053)		–			0.015	(0.205)
Age squared (CG)	−0.000*	(0.031)		–			0.000	(0.144)
Female	0.071	(0.201)		–			0.095	(0.057)
Care duration – (< 30 h)	(ref.)			(ref.)			(ref.)	
30 h–60 h	0.279***	(0.000)	0.06	0.258***	(0.000)	0.05	0.320***	(0.000)
60 h–150 h	0.465***	(0.000)	0.10	0.436***	(0.000)	0.09	0.450***	(0.000)
> 150 h	0.727***	(0.000)	0.17	0.710***	(0.000)	0.16	0.749***	(0.000)
Use of training	0.427***	(0.000)	0.09	0.454***	(0.000)	0.09	0.414***	(0.000)
Use of support group	0.235	(0.076)		0.296*	(0.015)	0.06	0.316**	(0.007)
Health status (CR) ^*a*^	0.194*	(0.016)	0.04	–			0.171*	(0.016)
More than one Disease (CR)	0.136*	(0.021)	0.03	0.137*	(0.011)	0.03	0.120*	(0.021)
Formal care (CR)	0.022	(0.710)		–			−0.020	(0.729)
ADL (CR)	0.032	(0.627)		–			0.036	(0.578)
IADL (CR)	−0.025	(0.659)		–			−0.010	(0.836)
MHI5 (CR)	0.002	(0.081)		0.002*	(0.032)	0.001	0.002	(0.109)
Constant	−3.435***	(0.000)		−2.507***	(0.000)		−2.946***	(0.000)
Number of observations	4033			4711			6201	

Notes: *p*-values in parentheses; Source: Capacites, Aides et REssources des seniors (CARE) – 2015; * *p* < 0.05, ** *p* < 0.01, *** *p* < 0.001; *M.E*. stands for marginal effects; *CG* informal carer; *CR* care recipient; ^a^ included the categories: Fair, bad and very bad (as reference, Very good; and Good)

**Fig. 2:**
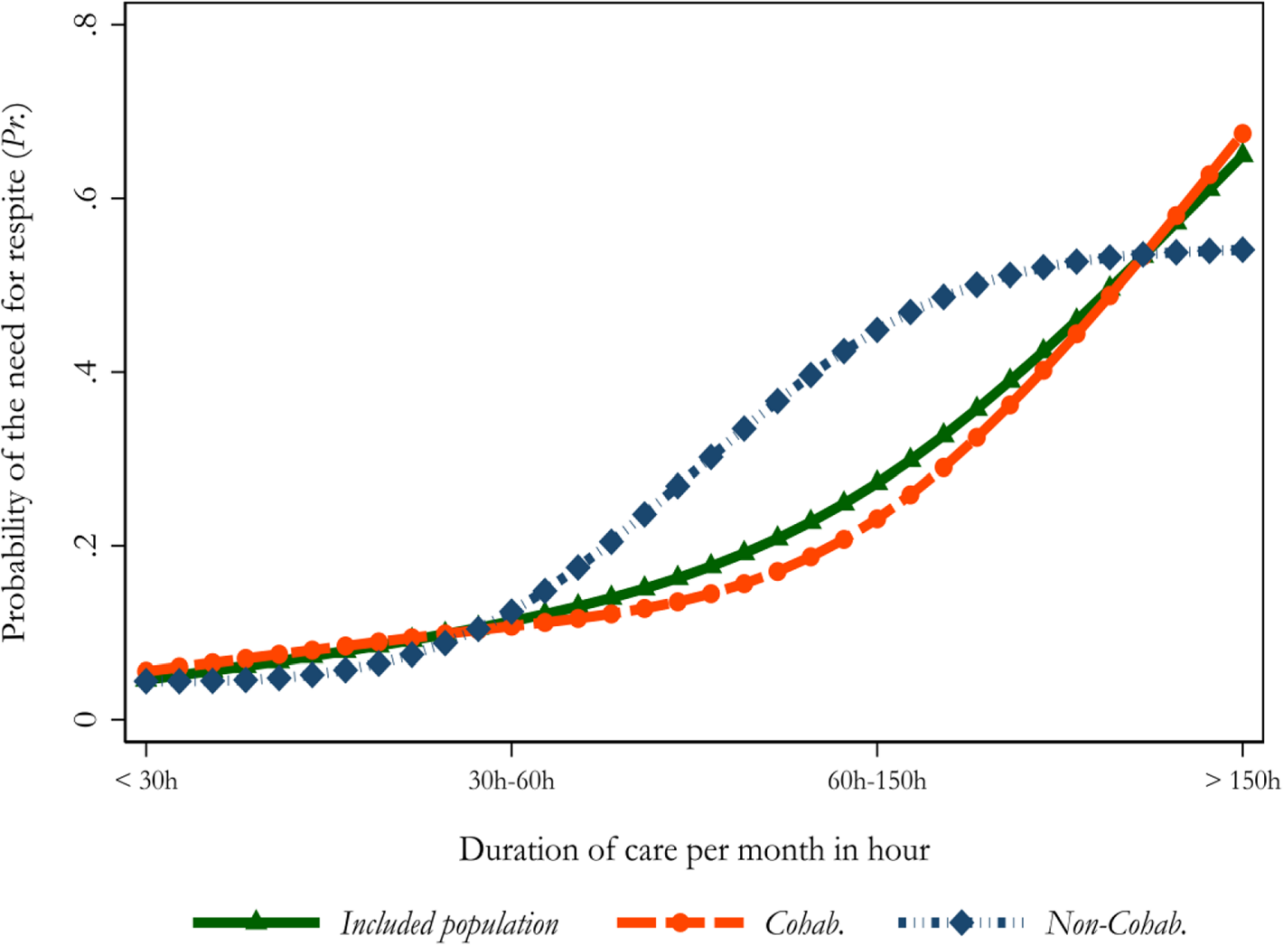
Probability of the need for respite according to the duration that carers had been providing support to care recipients

There was a higher need for respite for carers with the raise of the duration of care provided to recipients (*p* < 0.001). Figure 2 illustrates this finding.

Finally, regarding the characteristics of care recipients, carers’ need for respite increases with the worsening of the health status of the recipients (*p* < 0.05). Additionally, care recipients suffering from more than one chronic disease were more likely to create a higher need for respite (*p* < 0.05).

#### Secondary analysis

The results of the secondary analysis are reported in the appendix. The higher the strain (HSCI), the higher the need for respite (*p* < 0.001). As in the primary analysis, the other explanatory variables were also significant in secondary analysis. The filiation variable was significant, with a positive effect for children and family members for carers (*p* < 0.05). At the same time, the close acquaintance was less likely to need respite (*p* < 0.05). The more carers reported the problem of lack of time (*p* < 0.001), and the feeling of loneliness (*p* < 0.001), the higher the probability of the need for respite. The care duration (*p* < 0.001), the use of training (*p* < 0.001), and the use of support group (*p* < 0.05) positively affected the need for respite. The chronic condition of care recipients significantly increased the need for respite (*p* < 0.05).

## Discussion

This study is based on a very recent nationwide database *CARE*, collected in 2015. From this point of view, it brings new and additional information to the published work of this relationship given the steady increase in the number of old-aged people and carers and the lack of quantitative research into the determinants of the need for respite in France [25, 44, 48, 56, 63].

In this study, we used two different factors that relate to health: the health status of carers; and a measure of health status through a composite index (HSCI). We did this on purpose because these measures differ both in content and in measurement and focus on different but complementary aspects of health. The rationale in the realization of two separated models or analysis (primary/secondary) is particularly avoiding the problem of multicollinearity. Econometric modelling provides the finding that the health of carers is one of the most important determinants of the need for respite. The results of both analyses were relevant and quite similar in the sense that health status and HSCI turn out to be significant and had the same expected positive effect in explaining the need for respite. Thus, when informal carers experience poor health or strain, the need for respite increased. This finding could be explained by the fact that the negative consequences of informal care and its emotional impact widely affect carers’ health [1]. However, one should be cautious with international comparisons of the results, as research showed that cultural values might vary considerably within and between countries.

Nevertheless, regardless of the cultural values or living status of carers, the association between the health status of carers and the need for respite seems evident and intuitive. Our findings chime with those of Gervès-Pinquié et al. (2014), who found that the need for respite was associated with the health of carers in France. Some studies show that the need for respite depends not only on the health of carers and recipients but also on other carers characteristics. These findings accord with those reported in the Netherlands using data collected among Dutch informal carers *(respectively N = 950 and N = 273)* [25, 56] and in the U.S. based on community and informal carers survey data *(N = 1058)* [48].

Our findings also highlight that the living arrangement of carers with care recipients could significantly increase the probability of the need for respite. According to Schulz and Beach, certain forms of living arrangement increased the need for respite by 20% [30]. However, the assumption that carers living with their care recipients experience a considerable burden and require more respite has been confirmed empirically [57].

Carers not living with their recipient may have experienced less burden and less need, for instance. This result could be due to less informal care, considering the median volume of assistance provided by cohabiting carers is twice as high as that of non-cohabiting carers [64, 65]. However, in the multivariate analysis, after adjustment for the duration of informal support, the effect of cohabitation remains an independent factor. In other words, the presence around the immediate environment with the recipient could represent a mental burden for the carer. There is a lack of various respite interventions in the sense that some carers are not always aware of the availability of services. It sometimes appears that carers choose not to use support partially as a result of having difficulty accessing and using it and partially as a result of a lack of information [66–68].

Given the negative effect of the length of time providing care on the need for respite, it clearly appears that, in the case of a lack of respite assistance, informal carers, particularly those cohabiting with care recipients, have a sense of being stuck in a “trap” or they no longer need support. Therefore, it could not be at all surprising that some carers reported having renounced the need for respite mainly expressed by the feeling of reluctance [69]. Nonetheless, there is still a need for empirical evidence to support the assumption of the length of time providing care. Mostly, the feeling of guilt could discourage family members who are providing care from asking for in-home support for a relative [41]. Conversely, the duration for caregiving may likely increase the need for respite. This result is quite intuitive in the sense that the volume of informal care could create a greater burden for carers, independently of their living status [70].

Moreover, considering the intergenerational relationship between informal carers and recipients, the situation is complicated because some children are generally not willing to provide care to old-aged dependants, even though they represent the majority of informal carers [64, 71]. At the same time, the majority of old-aged dependants need assistance with daily activities [72]. In addition to this, the lack of time and the feeling of loneliness also determine the need for respite. Once again, this finding could be explained by a reluctance to place their care recipient in a support service in order to spend some time away [69].

Importantly, it has been shown that psychological, emotional responses to chronic diseases and illness of care recipients may sometimes be managed very poorly by informal care providers. This could reflect on carers the problem of the lack of training and awareness among those assuming a caregiving role, but also the fact that cultural values may differently affect informal carers.

Therefore, if policymakers want to rely on informal care as an essential input in health care in long-term care, they should first keep a close watch on the human capital, particularly on carers’ health [73, 74]. Following this, policymakers should identify strained carers at risk with high volume care through supervised learning to prevent carer exhaustion [75]. Since the French health system cannot necessarily accommodate all formal and informal costs related to carer and recipient health, there is a need for tailor-made respite, psychological and training/support programmes based on carers’ preferences. However, mixed evidence of the effectiveness of existing interventions should assist health policymakers [76–79]. Additionally, we find that the majority of informal carers (73%) who don’t need respite are less affected by the burden of care, compared to those who need respite. From this point, health policymakers could nationally prioritise the implementation of various support program to alleviate the burden of carers with a high volume of care when assisting older persons living with chronic afflictions. In all cases, it is important to prevent the high risk of exhaustion due to caregiving which could generate depression, anxiety, and/or other diseases. This study helped to better characterised the carer situation and to focus on major factors associated with the increasing need for respite for informal carers and to identify high risk groups of informal caregivers. A short questionnaire could be added, for example, in the framework of care recipient consultation and/or hospitalisation in order to assess if a carer is at high risk. If it is determined that they are at risk and if necessary, health screening, educational program or personalised training could be offered to the carer in order to protect carers’ health as far as possible and avoid the need for respite care. Our results could also lead to the implementation of a more personalised support plan for family members [80]. Further development could be based on data mining method applications [81].

Some limitations were identified in our paper. First, there is no denying that econometric modelling gives rise to the endogeneity problem for at least two primary sources. One possible source of endogeneity is the two-way (reverse) causality between the need for respite and the health status. Another possibility is the unobserved individual heterogeneity. Informal carers can differently report their experiences of strain because of higher levels of impairment. As a result, some exogenous factors such as age, relationship and/or gender may induce informal carers to provide incorrect estimates of the negative impact of care. Second, the dependent variable reflects a dichotomous measure (“yes” or “no”) of the need for respite. It has not been possible to assess the intensity on the preferences of carers (“never” or “sometimes” or “more often”). Third, future studies will want to analyse the demand for various supplies of support (respite) services and access how the characteristics of each carer enable or limit the preferences of carers. The utility function or behavioural model of informal carers towards the need for respite should be assessed in further investigations.

## Conclusions

Our study consists of an analysis of the data of the National French *CARE* survey, conducted in 2015. We show that the need for respite varies according to the characteristics of informal carers, as well as care recipients’ factors. Health status, feeling of loneliness, lack of time, care duration more than 30 h per month, use of training for informal carers and the number of chronic diseases of care recipients were the most significant characteristics impacting on the need for respite. This study has important health policy implications for the current ageing population crisis in the most OECD countries to prevent the high risk of exhaustion. A prioritisation scheme for policymakers could consist of conducting prevention policies to identify at-risk carers. Practically, the challenge for policymakers, physicians and other health professionals is to refer to the right service, e.g. psychological interventions, respite care support, training support, education support. Dealing with the dyad’s quality of life, innovative interventions that aimed to provide support to carers and/or carers’ family members have to be developed. However, there is a need for more economic evaluations of respite interventions for carers to assist policymakers in prioritising carers support programmes.

## Supplementary Information

**Additional file 1.**

## Data Availability

The data that support the findings of this study are available at in the website of the French National Institute for Statistics and Economic Studies (INSEE) and the Directorate for Research, Studies, Assessment and Statistics (DREES - French Ministry of Health): (https://drees.solidarites-sante.gouv.fr/etudes-et-statistiques/open-data/personnes-agees/article/les-enquetes-capacites-aides-et-ressources-des-seniors-care).

## Abbreviations

ADL: Activities of daily living
IADL: Instrumental activities of daily living
CARE: Capacités aides et ressources des seniors
DREES: Directorate for research, studies, assessment and statistics
HSCI: Health status composite index
IACO: International alliance of carer organizations
INSEE: National institute for statistics and economic studies
MHI5: Mental health index
OECD: Organisation for economic co-operation and development
PCA: Principal components analysis
SD: Standard deviation
