# Supplementary material for: Determinants of the need for respite according to the characteristics of informal carers of elderly people at home: results from the 2015 French national survey
Source: BMC Health Serv Res. 2021 Sep 21;21:995. doi: 10.1186/s12913-021-06935-x (PMC8456521; doi:10.1186/s12913-021-06935-x)
Supplement: Supplementary file 1 — Additional file 1. [file 12913_2021_6935_MOESM1_ESM.docx]

**Supplementary analysis**

**Appendix 1: Secondary analysis - Determinants of the need for the respite of informal carers**

|  |  | (1) | | (2) | | (3) | |
| --- | --- | --- | --- | --- | --- | --- | --- |
|  |  | Full model | | Model with stepwise backward selection | | Full model with multiple imputations | |
| Variables | | Coef. | p-value | Coef. | p-value | Coef. | p-value |
| HSCI | | 0.168*** | (0.000) | 0.171*** | (0.000) | 0.153*** | (0.000) |
| Length of time for care - (<1 year) | | (ref.) |  | (ref.) |  | (ref.) |  |
|  | 1 - 5 years | -0.094 | (0.438) | - |  | -0.105 | (0.369) |
|  | > 5 years | -0.137 | (0.250) | - |  | -0.108 | (0.387) |
| Income Level – (< 800€) | | (ref.) |  | (ref.) |  | (ref.) |  |
|  | 800€ - 1,200€ | 0.214 | (0.218) | - |  | 0.130 | (0.456) |
|  | 1,200€ - 1,800€ | 0.228 | (0.143) | - |  | 0.173 | (0.289) |
|  | 1,800€ - 2,500€ | 0.252 | (0.100) | - |  | 0.125 | (0.419) |
|  | > 2,500€ | 0.352* | (0.019) | - |  | 0.283 | (0.062) |
| Marital status – (Single) | | (ref.) |  | (ref.) |  | (ref.) |  |
|  | Married | -0.055 | (0.501) | - |  | -0.053 | (0.475) |
|  | Divorced | 0.183 | (0.208) | - |  | 0.294* | (0.019) |
|  | Widowed | -0.137 | (0.212) | - |  | -0.053 | (0.608) |
| Filiation – (Partner by marriage) | | (ref.) |  | (ref.) |  | (ref.) |  |
|  | Child | 0.195 | (0.074) | 0.239** | (0.003) | 0.202* | (0.034) |
|  | Family member | 0.275* | (0.024) | 0.298** | (0.003) | 0.234* | (0.027) |
|  | Close acquaintance | -0.436* | (0.043) | -0.424* | (0.023) | -0.489* | (0.045) |
| Care for other persons | | 0.133* | (0.040) | 0.151* | (0.013) | 0.119* | (0.030) |
| Cohabitation | | 0.187* | (0.026) | 0.175* | (0.019) | 0.141 | (0.054) |
| Feeling of loneliness | | 0.269*** | (0.000) | 0.245*** | (0.000) | 0.267*** | (0.000) |
| Lack of time | | 1.206*** | (0.000) | 1.232*** | (0.000) | 1.193*** | (0.000) |
| Replacement in case of unavailability | | -0.045 | (0.473) | - |  | -0.111 | (0.063) |
| Age (CG) | | 0.028* | (0.035) | - |  | 0.017 | (0.143) |
| Age squared (CG) | | -0.000* | (0.026) | - |  | 0.000 | (0.120) |
| Female | | -0.046 | (0.426) | - |  | 0.000 | (0.997) |
| Care duration – (<30h) | | (ref.) |  | (ref.) |  | (ref.) |  |
|  | 30h-60h | 0.248** | (0.001) | 0.239*** | (0.001) | 0.310*** | (0.000) |
|  | 60h-150h | 0.413*** | (0.000) | 0.387*** | (0.000) | 0.411*** | (0.000) |
|  | >150h | 0.639*** | (0.000) | 0.626*** | (0.000) | 0.673*** | (0.000) |
| Use of training | | 0.369*** | (0.000) | 0.378*** | (0.000) | 0.355*** | (0.000) |
| Use of support group | | 0.181 | (0.193) | 0.252* | (0.049) | 0.276* | (0.023) |
| Health status (CR) *^a^* | | 0.159* | (0.047) | - |  | 0.127 | (0.073) |
| More than one Disease (CR) | | 0.154* | (0.011) | 0.155** | (0.005) | 0.131* | (0.013) |
| Formal care (CR) | | 0.008 | (0.896) | - |  | -0.027 | (0.643) |
| ADL (CR) | | 0.048 | (0.482) | - |  | 0.048 | (0.467) |
| IADL (CR) | | -0.037 | (0.516) | - |  | -0.019 | (0.709) |
| MHI5 (CR) | | 0.002 | (0.083) | 0.003* | (0.029) | 0.002 | (0.116) |
| Constant | | -3.151*** | (0.000) | -2.246*** | (0.000) | -2.696*** | (0.000) |
| Number of observations | | 4,038 | | 4,716 | | 6,201 | |

*Notes: p*-values *in parentheses; Source: Capacites, Aides et REssources des seniors (CARE) – 2015;* * p<0.05, ** p<0.01, *** p<0.001*; CG: informal carer; CR: care recipient; ^a^ included the categories: Fair, bad and very bad.*
